# Harnessing laboratory data for poliovirus eradication: contributions of the Africa regional polio laboratory data management team, 2022 – 2024

**DOI:** 10.1016/j.cmpbup.2025.100214

**Published:** 2025

**Authors:** Brook Tesfaye, Reggis Katsande, Doungmo Wakem Yannick Arthur, Julius E Chia, Chefor Ymele Demeveng Derrick, Ikeonu Obianuju Caroline, Kabore Sakma, Mahmud Zubairu, Busisiwe Ngobe, Abdulahi Walla Hamisu, Ticha Johnson Muluh, Kebba Touray, Modjirom Ndoutabe, Jamal A Ahmed, Anfumbom Kfutwah

**Affiliations:** Polio Eradication Program, Office of the Regional Director, World Health Organization, Regional Office for Africa, Brazzaville, Republic of the Congo

**Keywords:** Poliovirus eradication, Africa polio laboratory network (APLN), Data management, Surveillance, Data use, Data quality

## Abstract

**Background and Objectives:**

Polio laboratory data is crucial in providing timely and accurate information on poliovirus outbreaks and therefore an important component of the overall poliovirus eradication strategies. This paper discusses the contributions of the Africa Regional Polio Laboratory Data Management Team (RPLDMT) in optimizing data-driven polio eradication efforts in the African region from 2022 to 2024.

**Methods:**

We explored key data management activities performed by the RPLDMT from 2022 to 2024 and assessed their contribution on enhancing polio eradication efforts in the African region.

**Results:**

The RPLDMT has significantly advanced polio eradication efforts in Africa through multiple initiatives. Notably, the team has supported the Africa Regional Emergency Operations Center (EOC) by providing 218 daily line lists of polioviruses identified, improving real-time case tracking and decision-making. The integration of Open Data Kit (ODK), an open-source electronic data collection tool, has enhanced poliovirus environmental surveillance, benefitting 23 countries in 2022, 13 in 2023, and 14 as of August 2024. The development of a sophisticated automated data quality assurance script has improved data accuracy and reliability, with 65 weekly line lists of errors provided for data correction. Additionally, the introduction of the biweekly Africa Regional Polio Laboratory Network (ARPLN) bulletin and real-time dashboards has optimized data use, aiding in actionable insights and decision-making. Efforts to transition to the Web-based Information for Action (WebIFA) system and capacity building through training workshops have further strengthened data management and surveillance capabilities across the region.

**Conclusion:**

The contributions provided by the RPLDMT has played a key role in boosting the polio eradication efforts with a focus on enhancing human resource skills embracing new technologies and implementing real-time performance monitoring tools to improve data quality and strengthen data-driven decision-making processes essential for speeding up the progress towards eradicating polio in the region.

## Highlight

• Discusses the contributions of the Africa Regional Polio Laboratory Data Management Team (RPLDMT) in optimizing data-driven polio eradication efforts in the African region.
• Presents polio laboratory data quality assurance techniques implemented.
• Presents the use of data for decision making in polio eradication program.
• Discusses activities implemented in strengthening polio laboratory information systems in the African region.
• Discusses the application of technology in enhancing polio surveillance initiatives.
• Shows training and capacity building activities of Data management officers.

## 1 Introduction

Since its establishment in the 1988, the Global Polio Eradication Initiative (GPEI) has made significant strides in reducing the transmission of poliomyelitis, successfully vaccinating over 2.5 billion children. This initiative has resulted in a drastic decline of over 99 percent in cases of Wild Poliovirus (WPV), decreasing from an estimated 350,000 cases in 1990 to 44 cases as of August 2024 [1]. Currently, only two countries, Afghanistan and Pakistan, remain endemic for WPV. However, despite the advancements made in global poliovirus eradication efforts, several challenges persist [2].

In August 2020, a significant achievement in global health was achieved when the Africa Regional Certification Commission (ARCC) officially declared that Africa is free of WPV [3]. Nevertheless, the fight against polio continues to face challenges most particularly driven by socio-economic and security factors coupled with suboptimal primary healthcare programs and significantly low routine immunization coverage. Moreover, some countries in the African region particularly the Democratic Republic of Congo (DRC) and Nigeria continue to experience outbreaks of circulating Vaccine-derived Poliovirus (cVDPV) underscoring the necessity for tailored approaches to enhance vaccination efforts and overcome barriers to eradication [[4], [5], [6]]. These approaches can include engaging local leaders for community engagement and addressing vaccine hesitancy, utilizing fractional dose vaccination where vaccine supply is limited, empowering female health workers, implementing robust surveillance system and vaccination campaigns security compromised areas [6,7].

Consequently, the GPEI is dedicated to enhancing Acute Flaccid Paralysis (AFP) surveillance, bolstering routine immunization programs, and improving the quality and timeliness of outbreak response in the African region. Furthermore, it is essential to learn lessons from past challenges such as the Coronavirus Disease (COVID-19) pandemic to enhance public health initiatives, particularly by emphasizing the need to incorporate polio eradication strategies into broader initiatives aimed at strengthening primary healthcare systems including routine immunization [5,6].

The Global Polio Laboratory Network (GPLN), created in the 1980s as a component of the GPEI, is vital in delivering prompt and accurate laboratory findings to support global polio surveillance and eradication efforts. The GPLN consists of 146 polio laboratories accredited by the World Health Organization (WHO) across 92 countries, organized into National Laboratories (NLs), Regional Reference Laboratories (RRLs) and Global Specialized Laboratories (GSLs) [8]. The Africa Polio Laboratory Network (APLN) is a subset of the GPLN, and it includes 16 laboratories supported by the WHO.

The Regional Polio Laboratory Data Management Team (RPLDM) overseas the systematic collection, analysis, and reporting of data from polio laboratories. The data collected using AFP Case Investigation Form (CIF) and the environmental surveillance sample collection form [9] is then entered into a polio laboratory information system, developed using Epi info software with a Microsoft (MS) Access backend [10]. Efficient data management includes timely result reporting, data quality audits, and the use of electronic data tools to enhance stakeholder communication [10]. Objective 5 of the Global Polio Surveillance Action Plan (GPSAP) for 2022 to 2024 emphasizes on enhancing the sensitivity of polio surveillance by improving data collection techniques through technology integration and fostering collaboration, among stakeholders [11]. It also emphasizes data accuracy, quality, and completeness critical for tracking and eradicating polio globally [11].

Despite progress, the APLN faces challenges in data quality and its utilization for program needs. Strengthening data analysis, improving laboratory staff skills, and reinforcing information management systems are essential for effective decision making and achieving polio eradication goals.

The RPLDMT has played a key role in enhancing laboratory data management in the African region by establishing standards and protocols, ensuring data quality, promoting data-driven action, and maintaining an up-to-date laboratory database. This paper discusses RPLDMT’s contributions in improving polio laboratory data quality and utilization to optimize eradication efforts in the African region from 2022 to 2024.

## 2 Methods

### 2.1 Overview of the Africa Polio Laboratory Network (APLN)

**Fig. 1:**
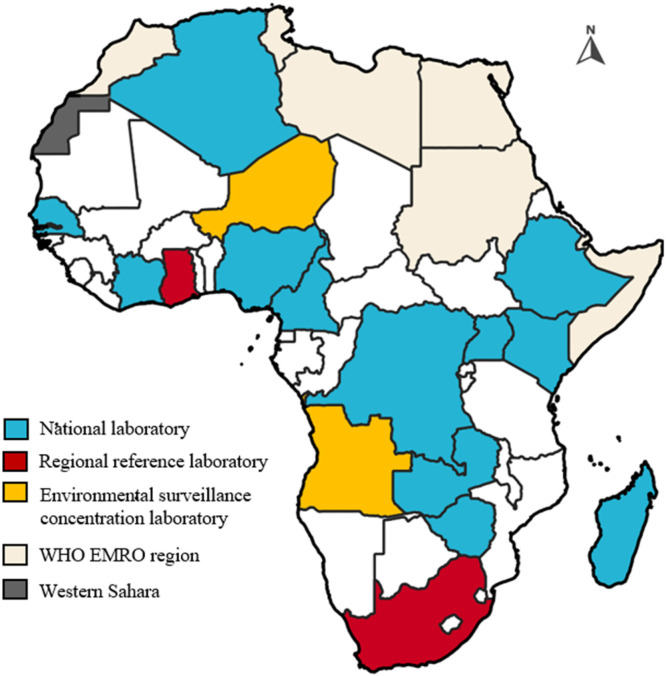
The Africa Polio Laboratory Network (APLN), 2024.

The APLN is a part of the GPLN consisting of 16 WHO-supported laboratories dedicated to detecting and tracking polioviruses from both stool and environmental samples throughout the region. This helps to pinpoint poliovirus circulating areas even in the absence of clinical cases and generates data that guide programmatic and vaccination activities [8]. Recent advancements in the APLN include setting up Zimbabwe polio laboratory to test sewage samples for polioviruses, and additionally, polio laboratories in Nigeria, Cote d’Ivoire, Madagascar, and Zambia undergone major upgrades, such as installing new equipment and improving facilities to enhance testing capacity. Despite these advancements, the network faces challenges like growing workloads and limited resources. To sustain efficiency and effectiveness in fighting polio across the African region, further investment in infrastructure and workforce expansion is essential. Fig. 1 depicts the polio laboratory network in the African region, categorized by their functions, including national laboratories (laboratories performing basic poliovirus isolation procedures) and regional reference laboratories (laboratories performing genomic sequencing). Many of these laboratories support multiple countries beyond their host country, providing essential services across the region.

**Fig. 2:**
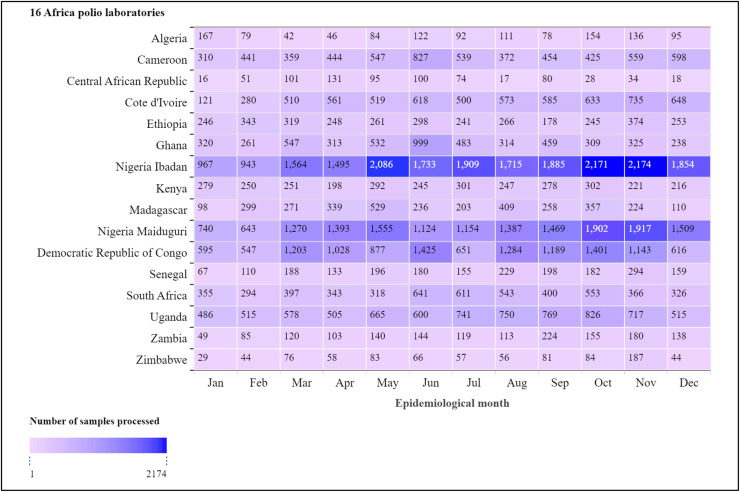
Sample workload analysis of the number of AFP samples processed by the 16 Africa polio laboratories in the African region, January to December 2023 (*N* = 94,752).

The RPLDMT, a central component of this network, is responsible for the daily management and reporting of polio laboratory data produced from all these laboratories, essential for timely and accurate decision-making and public health responses. Fig. 2 presents sample workload analysis of the number of stool samples analyzed by the 16 Africa polio laboratories in the African region, from January to December 2023. From the data, the two polio laboratories in Nigeria consistently analyzed the highest number of samples. Other polio laboratories in Democratic Republic of Congo. Uganda, and South Africa also had relatively high sample processing volumes.

### 2.2 The Africa Polio Laboratory Data Management Team (RPLDMT) and Terms of Reference (ToR)

The RPLDMT comprises dedicated professionals, each bringing unique expertise to the data analysis and management in the Polio Eradication Program (PEP) in Africa. The team, which successfully completed a one-week pre-deployment specialized training in March 2022 on routine immunization, AFP, and other Vaccine Preventable Diseases (VPDs) surveillance data management, is based at the regional WHO office in the Republic of Congo, Brazzaville.

The ToR was developed considering the GPEI core strategy of increasing efficiency in collecting, managing, and using data for action as outlined in objective five [11] of the GPSAP focusing on providing technical assistance to member states, polio laboratories, and inter-country teams towards data-driven polio eradication. The ToRs therefore included but are not limited to 1) strengthening data management activities in the Africa region and providing technical support to member states; 2) fostering the application of technology and integrating innovation in polio eradication initiatives; 3) Providing technical support to National and regional polio laboratories and; 4) coordination and collaboration with governments, technical partners, other WHO regions and WHO headquarters.

### 2.3 Africa regional polio laboratory data flow

**Fig. 3:**
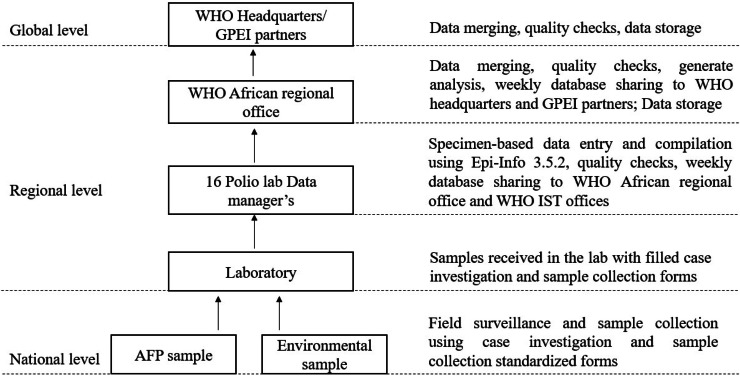
Overview of the regional polio laboratory data flow, 2024.

The flow of poliovirus data begins with the detection of AFP cases or the collection of environmental samples (Fig. 3). Standardized data collection is facilitated using the AFP CIF and the environmental surveillance sample collection form [12]. The responsibility for entering data related to laboratory components of samples received from the field into the polio laboratory database lies with the 16 polio laboratory data managers. This database, built on Microsoft Access is populated using a custom data entry form developed with Epi-info statistical software (version 3.5.2; CDC, Atlanta, USA), serves as a critical tool for data management [12].

Every Friday before end of the business day, the 16 data managers share their database with the WHO Africa Regional Office. Data quality, timeliness, and completeness verifications are carried out by three data managers assigned to WHO inter-country support teams [12]. This step is crucial for maintaining the accuracy and reliability of the information shared. Subsequently, the regional data management team at WHO AFRO compiles and disseminates a comprehensive regional database to member states, WHO headquarters, and other technical partners every Monday. This system ensures efficient data management, sharing, and analysis, which are essential for monitoring poliovirus transmission and guiding outbreak response strategies.

### 2.4 Data analysis and presentation

For data management, analysis, and presentation, R programming language for windows version 4.3.1 and Quantum GIS version 3.24.1 are utilized. The polio laboratory database for 2023 was used to highlight the number of samples processed by the 16 polio laboratories in Africa.

### 2.5 Ethical considerations

Not applicable.

## 3 Results and discussion

### 3.1 Contribution to the Africa regional Emergency Operations Center (EOC)

The Africa Regional Emergency Operations Center (EOC), led by the PEP coordinator at the WHO regional office, is dedicated to overseeing and organizing efforts to combat poliovirus outbreaks and advance polio eradication initiatives throughout Africa. The RPLDMT assists the EOC by providing a daily listing and reporting of confirmed poliovirus notifications. The data presented in the line list of confirmed poliovirus cases, disaggregated by country, province, and district, were used to inform key decisions such as planning vaccination campaigns, deployment of vaccines in response to confirmed cases, resource allocation, and monitoring efforts. The data guided the identification of areas requiring immediate intervention and helped allocate available funding to high-priority regions. Thus, this data facilitates rapid, informed decision-making for the Polio eradication initiative in the Africa region led by the PEP [13].

The line list is a detailed and systematic spreadsheet that records individual confirmed poliovirus cases which provides a comprehensive and organized way to track and analyze case-specific data. It compiles information on Epidemiological Identification (EPID) numbers, demography, dates on the onset of paralysis and sample collection, sample adequacy and shipment and the different laboratory results from virus isolation to sequencing.

From a data management perspective, the implementation of daily updates enables monitoring of poliovirus cases ensuring that any new cases or outbreaks are promptly reported and addressed. Regular updates of these data helps to maintain an up-to-date, complete, and accurate information on poliovirus circulations in the region. This is essential in taking data-driven action implementing targeted interventions in the affected areas and adjusting strategies as necessary to improve the efficiency and impact of eradication efforts. The findings of this study aligns with a previous publication made in Nigeria which discusses the role of real-time poliovirus data in guiding Nigeria’s EOC to make informed decisions regarding outbreak response activities, operations, and other coordination efforts [13]. From 9 October 2023 up to 22 August 2024, the RPLDMT has disseminated 218 daily line lists to the Africa regional polio EOC.

### 3.2 Technology and integrated innovations in poliovirus environmental surveillance

The RPLDMT plays an important role in providing technical assistance to member states in establishing an efficient, flexible, and reliable system for collecting and managing data. A significant part of this assistance involves the development of electronic data tools using the Open Data Kit (ODK) technology, a mobile-based data collection tool that has greatly improved the implementation of poliovirus environmental surveillance. By integrating ODK technology, the quality of data collected has improved, facilitating timely and evidence-based decision-making processes. ODK enhanced data quality by incorporating real-time validation, which ensures that only valid data is entered; skip and conditional logic, which tailor data collection forms based on previous responses, reducing irrelevant or inconsistent entries; and built-in consistency checks that validate data against predefined rules. Additionally, it enabled users to collect geographic data crucial for poliovirus field surveillance. Our results are consistent with other similar observations from Nigeria [14], Congo [15], and Kenya [16], which reported the crucial role of ODK technology in enhancing poliovirus surveillance and other polio eradication efforts in the Africa region emphasizing the validity of our contribution.

The RPLDMT has been actively involved in providing on-site support and guidance to promote the use of ODK technology. This effort has been rolled out with the support provided to >750 officers in 23 countries in 2022, 13 countries in 2023 and 14 countries as of 5 August 2024. Table 1 presents the various tools developed using ODK technology, their objectives, and the key information gathered through the development of these tools. This systematic approach not only reinforces monitoring but also ensures that data management practices are consistently improved throughout Africa.

**Table 1:** The application of ODK technology in environmental surveillance in the African region, 2024.

Name of the ODK tool	Objectives	Some key information collected using the tool	Progress as of 2024
Environmental surveillance site specification	To gather detailed descriptions and characteristics of Environmental surveillance sites.	GPS, site name, demographic data, site supervisor and sample collectors, date site opened, site type, sample collection frequency, catchment population, site photo.	Baseline site specification data have been successfully collected for 70 % of environmental surveillance sites in the African region, enhancing monitoring and site characterization efforts
Environmental surveillance site monitoring	To review the performance of the site, data collected with the form provides evidence required to keep a functional ES site open or to close a site that is underperforming in terms of poliovirus detection over a sustained period of time.	GPS, name, demographic data, site type, flow type, site quality, availability of high-risk population, the color of sewage, odor of sewage, availability of sample collection schedule, availability of Personal Protective Equipment (PPEs), availability of logistics for sample transportation, name of monitors, date site reviewed.	The site monitoring tool has been successfully deployed to 70 % of functional environmental surveillance sites, improving oversight and operational efficiency.
Environmental surveillance sample collection supervision	To supervise sample collection sessions in line with the SOPs.	Demographic data, site name, GPS, name of sample collectors and supervisors, EPID number, sample collection date and time, sample condition, adequate sewage flow rate, presence of chemical discharge.	74 % of the total environmental sampel collection schedules were supervised using sample collection supervision form in ODK
Digitize waterway	To collect the geo-coordinates of the waterway/stream from the environmental surveillance site point of collection (from downstream to upstream) to calculate the catchment area and its estimated population draining into the site's point of collection.	Site name, demographic data, site type, site flow type. Every 50 m a geo-coordinate is taken following the waterway of the site. This data will be used to plot the map and calculate the catchment population draining to the environmental surveillance site.	70 % of environmental surveillance sites have been successfully digitized using the Digitize Waterway ODK tool, improving site mapping and data accuracy.
Environmental surveillance country documentation	To provide evidence of the critical environmental surveillance activities being carried out in the country.	Demographic data, availability of environmental surveillance operational plan, total number of functional sites, presence of designated national focal point, training provided.	Among the 45 implementing countries, 75 % have successfully submitted records on environmental surveillance documentation, strengthening data completeness and program monitoring efforts.

### 3.3 Contribution to enhancing data quality

Polio data quality is crucial for the success of the GPEI, particularly in the African region, where the accurate collection and analysis of data are vital for tracking progress and shaping immunization strategies [17]. To address the challenges of data quality, the RPLDMT has developed a sophisticated automated data quality assurance script. This script, created using Notepad and executable in EpiInfo version 3.5.2, focuses on critical variables that are essential for computing key performance indicators used to monitor and guide polio eradication efforts in line with the GPSAP for 2022 – 2024 [11]. The script has been tailored for use with both AFP and environmental surveillance databases, enhancing the precision and reliability of the data collected.

**Fig. 4:**
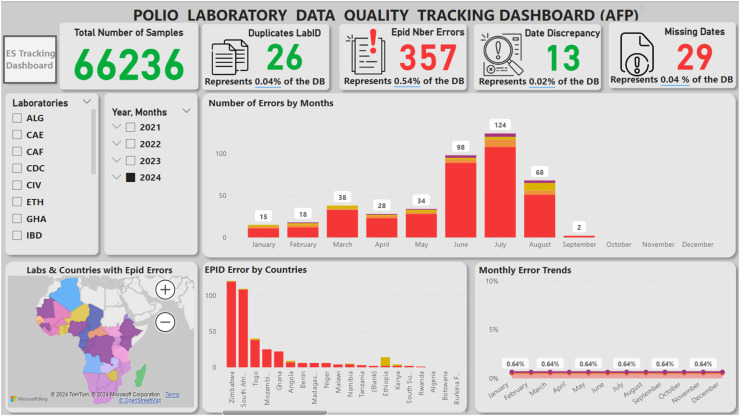
Real-time polio laboratory data quality tracking dashboard, 2024.

The RPLDMT utilizes the data quality assurance script to analyze data quality, generating automatic feedback in the form of line list for each of the 16 polio laboratory data managers in the Africa region, and from January 2023 up to 22 August 2024, the RPLDMT has disseminated 65 weekly line lists. These feedback are instrumental in identifying erroneous records, which are then corrected through a systematic follow-up process on a weekly basis. Furthermore, to provide a comprehensive view of progress at the regional level, a real-time dashboard has been developed using Microsoft Power Business Intelligence (MS Power BI). For example as depicted in Fig. 4, during the analysis time the polio laboratory data quality tracking dashboard shows that a total of 66,236 samples have been processed. The dashboard reveals data quality issues including 26 duplicate LabIDs, 357 EPID number errors, 13 records with date discrepancies, and 29 records with missing dates. This dashboard facilitates ongoing tracking and enables stakeholders to make data-driven decisions, ultimately contributing to the effectiveness of polio eradication efforts in the region. Our study findings are similar to the conclusions drawn by Ntsama et al. [12], which emphasized the importance of consistent feedback to improve the quality of polio data in terms of timeliness, completeness, and consistency.

### 3.4 Contribution to optimizing data use for action

**Fig. 5:**
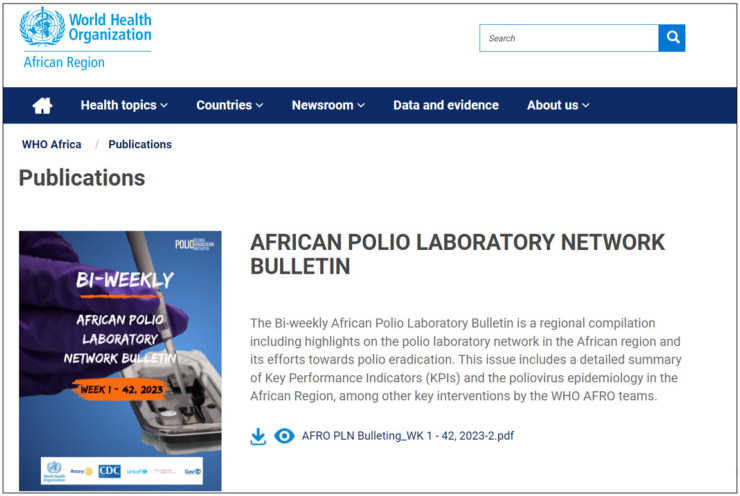
The Africa Polio Laboratory Network (APLN) bi-monthly bulletin published on the WHO African Regional Office (AFRO) official website, 2024.

Promoting the use of data for action in polio eradication requires harnessing data and insights to drive effective interventions and decision-making. To enhance the utilization of polio laboratory data, the RPLDMT has introduced the biweekly African Regional Polio Laboratory Network (ARPLN) bulletin. This bulletin leverages advanced data analysis and visualization techniques, focusing on over 10 key performance indicators designed to track core laboratory activities and assess intervention effectiveness. Distributed to >150 professionals, including those within the APLN, WHO headquarters, and various GPEI technical partners, the bulletin is shared via email and published online on various platforms such as Reliefweb (https://reliefweb.int/report/algeria/african-polio-laboratory-network-bulletin-week-1–42–2023) and the WHO African Regional Office official website (https://www.afro.who.int/publications/african-polio-laboratory-network-bulletin) (Fig. 5). It plays a crucial role in translating extensive laboratory data into actionable insights, serving as a robust feedback mechanism that aids laboratories in performance tracking and develop interventions for continuous improvement.

In addition to various ad-hoc data analyses and information production, the RPLDMT routinely produces technical and performance reports on poliovirus surveillance situations and conducts regular desk reviews. A key component of this effort is the quarterly poliovirus environmental surveillance desk review, which plays a vital role in enhancing data use within the context of polio eradication. The desk review provides a detailed analysis using six key performance and process indicators in line with the GPSAP for 2022 – 2024 [11] across all environmental surveillance implementing countries in the African region. By offering a comprehensive overview, the desk review supports informed decision-making and aids in the development of targeted interventions and strategies for polio eradication. It ensures that resources are effectively allocated to areas of greatest need, thereby enhancing program impact. Ultimately, the insights from these desk reviews enable program managers and policymakers to make evidence-based decisions that improve intervention effectiveness, and polio immunization service delivery, and contribute significantly to the goal of eradicating polio and addressing other public health challenges.

**Fig. 6:**
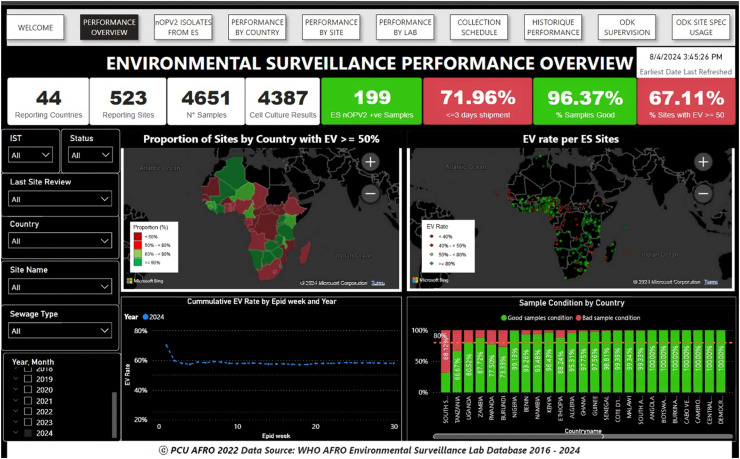
Real-time poliovirus environmental surveillance performance monitoring dashboard, 2024.

Data visualization dashboards significantly enhance data use by transforming complex datasets into clear, intuitive, interactive, and actionable insights [18,19]. The regional poliovirus environmental surveillance dashboard (https://app.surveillance performance tracking dashboard) has been capable of condensing vast amounts of data into concise visual summaries, allowing users to quickly grasp real-time or near real-time insights and essential information without delving into raw data enabling timely decision-making. Users can interact with the data by drilling down into specifics, filtering countries and years, and adjusting additional parameters, which helps in analyzing and triangulating different aspects of the data and making informed decisions (Fig. 6). Moreover, the dashboard increased engagement, communication, and data accessibility.

### 3.5 Contribution to strengthening the polio Laboratory Information System (LIS)

Efficient collection and processing of reliable surveillance data are crucial for the GPEI. The GPEI Strategy advocates for a transition to web-based information systems and electronic mobile data collection tools, where feasible, to enable real-time monitoring of virus detection. In the Africa region, while Epi Info remains widely utilized, teams frequently rely on a combination of MS Access databases and MS Excel for managing AFP and environmental surveillance data. However, these tools present limitations, hindering the ability to perform real-time data collection, reporting, and tracking of specimens from the field to the laboratory. To address these challenges, the region initiated and is near completion to transitioning from the Epi Info-based information for action system to a web-based Information for Action (WebIFA) system. This new system, has been successfully piloted in Afghanistan and Pakistan [11]. WebIFA integrates laboratory and field data into a single platform, facilitating real-time data exchange by consolidating information on AFP cases, contacts, specimens with laboratory results, environmental site data, and samples. The RPLDMT in collaboration with WHO headquarters and other technical partners and the regional PEP’s data and information management unit, is actively coordinating and participating in the development and implementation of WebIFA in the Africa region. To facilitate seamless transition from the current Epi Info to the WebIFA for the WHO Africa region, all 16 laboratories’ data managers and the regional office focal persons have been trained on WebIFA.

### 3.6 Contribution to regional human resource capacity building

Between 2022 and 2024, there were concerted efforts to improve the skillset of polio laboratory data managers in the African region through two annual training workshops. The first training workshop took place in Abuja, Nigeria from May 8 to May 12, 2023, followed by a second in Nairobi, Kenya from June 24 to June 28, 2024. These training workshops brought together a group of over 100 participants, including polio laboratory data managers, polio surveillance officers, WHO Inter Country Support Team (IST) office data managers, WHO headquarters, and various technical partners. The training aimed to address critical aspects of data management by focusing on improving data quality, utilizing data for actionable insights, and sharpening data manager’s technical skills in existing data management technologies and innovations in poliovirus surveillance and laboratory data management.

Following these successive trainings, the timeliness and completeness of polio laboratory data and reports have been improved by over 90 %, and laboratories have enhanced data utilization by producing programmatic bulletins and performance reports on key performance indicators to support informed decision-making.

A significant component of the training was dedicated to the implementation of the WebIFA system, ensuring that attendees gained practical skills and insights necessary for effectively managing and using polio laboratory data in their respective roles. Apart from these two annual training workshops the RPLDMT carried out regular on-site field visits, on-the-job training (OJT), and mentorship programs, at the 16 regional polio laboratories.

## 4 Conclusions

The RPLDMT has played and continues to play a valuable role, in enhancing polio eradication efforts in the region, 2022 to 2024. This includes transitioning towards a web-based polio laboratory information system, ensuring data quality through automated scripts, conducting training workshops, and creating real-time data and information visualization dashboards for decision-making. These initiatives have streamlined data collection processes, improved stakeholder coordination, and strengthened surveillance and laboratory systems. Additionally, the RPLDMT's focus on capacity building of key focal officers at country and regional levels through targeted training sessions and mentorship has significantly boosted the technical skills of polio laboratory data managers. These initiatives are a demonstration of the important role of data in providing programmatic guidance for achieving the much-needed certification of all forms of polioviruses for the WHO African region. As eradication plans advance, it is hoped that the RPLDMT will continue to be strengthened to support the region's initiatives effectively.

## Statements of ethical approval

Ethical clearance was not required for this study as it did not involve experimentation on human subjects or the use of identifiable personal data.

## Funding

This research did not receive any specific grant from funding agencies in the public, commercial, or not-for-profit sectors.

## CRediT authorship contribution statement

**Brook Tesfaye:** Data curation, Conceptualization, Writing – original draft, Visualization, Methodology, Formal analysis, Writing – review & editing. **Reggis Katsande:** Writing – review & editing, Supervision. **Doungmo Wakem Yannick Arthur:** Visualization, Writing – review & editing. **Julius E Chia:** Writing – review & editing. **Chefor Ymele Demeveng Derrick:** Writing – review & editing. **Ikeonu Obianuju Caroline:** Writing – review & editing. **Kabore Sakma:** Writing – review & editing. **Mahmud Zubairu:** Writing – review & editing. **Busisiwe Ngobe:** Writing – review & editing. **Abdulahi Walla Hamisu:** Supervision, Writing – review & editing. **Ticha Johnson Muluh:** Supervision, Writing – review & editing. **Kebba Touray:** Writing – review & editing, Supervision. **Modjirom Ndoutabe:** Writing – review & editing, Supervision. **Jamal A Ahmed:** Writing – review & editing, Supervision. **Anfumbom Kfutwah:** Supervision, Methodology, Writing – original draft, Validation, Writing – review & editing, Conceptualization.

## Declaration of competing interest

Authors declare no competing interests and conflict of interest.

## References

[1] Kishore N. (2024). Surveillance to track progress toward polio eradication—Worldwide, 2022–2023. MMWR. Morb. Mortal. Wkly. Rep..

[2] Lopez Cavestany R., Eisenhawer M., Diop O.M., Verma H., Quddus A., Mach O. (2024). The last mile in Polio eradication: program challenges and perseverance. Pathogens.

[3] Leke R.G.F., King A., Pallansch M.A., Tangermann R.H., Mkanda P., Chunsuttiwat S. (2020). Certifying the interruption of wild poliovirus transmission in the WHO African region on the turbulent journey to a polio-free world. Lancet Glob. Health.

[4] Amzat J., Razum O., Kanmodi K.K. (2023). Polio-philanthropy in Africa: a narrative review. Health Sci. Rep..

[5] Tediosi F., Villa S., Levison D., Ekeman E., Politi C. (2024). Leveraging global investments for polio eradication to strengthen health systems’ resilience through transition. Health Policy Plan.

[6] Deressa W., Kayembe P., Neel A.H., Mafuta E., Seme A., Alonge O. (2020). Lessons learned from the polio eradication initiative in the Democratic Republic of Congo and Ethiopia: analysis of implementation barriers and strategies. BMC. Public Health.

[7] Ezezika O., Mengistu M., Opoku E., Farheen A., Chauhan A., Barrett K. (2022). What are the barriers and facilitators to polio vaccination and eradication programs? A systematic review. PLOS. Glob. Public Health.

[8] Diop O.M., Kew O.M., de Gourville E.M., Pallansch M.A. (2017). The global polio laboratory network as a platform for the viral vaccine-preventable and emerging diseases laboratory networks. J. Infect. Dis..

[9] Poy A., Minkoulou E., Shaba K., Yahaya A., Gaturuku P., Dadja L. (2016). Polio Eradication Initiative contribution in strengthening immunization and integrated disease surveillance data management in WHO African region, 2014. Vaccine.

[10] Benke A., Williams A.J., MacNeil A. (2017). The stop transmission of Polio Data Management (STOP DM) assignment and its role in polio eradication and immunization data improvement in Africa. Pan. Afr. Med. J..

[11] Organization W.H. (2022).

[12] Ntsama B., Bwaka A., Katsande R., Obiang R.M., Oyaole D.R., Mkanda P. (2021). Polio data quality improvement in the African region. J. Immunol. Sci..

[13] Braka F., Adamu U., Siddique A., Bolu O., Damisa E., Banda R. (2023). The role of polio emergency operations centers: perspectives for future disease control initiatives in Nigeria. Pan. Afr. Med. J..

[14] Bammeke P., Erbeto T., Aregay A., Kamran Z., Adamu U.S., Damisa E. (2023). Assessment of open data kit mobile technology adoption to enhance reporting of supportive supervision conducted for oral poliovirus vaccine supplementary immunization activities in Nigeria, March 2017-February 2020. Pan. Afr. Med. J..

[15] Akpan G., Bello I., Mohamed H.F., Touray K., Kipterer J., Ngofa R. (2021). The digitization of active surveillance: an insight-based evaluation of interactive visualization of active case search for polio surveillance to support decision making in Africa. BMC. Public Health.

[16] Tesfaye B., K Makam J., Sergon K., Onuekwusi I., Muitherero C., Sowe A. (2020). The role of the stop transmission of Polio (STOP) program in developing countries: the experience of Kenya. BMC. Public Health.

[17] Scobie H.M., Edelstein M., Nicol E., Morice A., Rahimi N., MacDonald N.E. (2020). Improving the quality and use of immunization and surveillance data: summary report of the Working Group of the Strategic Advisory Group of Experts on Immunization. Vaccine.

[18] Dixon B.E., Grannis S.J., McAndrews C., Broyles A.A., Mikels-Carrasco W., Wiensch A. (2021). Leveraging data visualization and a statewide health information exchange to support COVID-19 surveillance and response: application of public health informatics. J. Am. Med. Inform. Assoc..

[19] Rabiei R., Bastani P., Ahmadi H., Dehghan S., Almasi S. (2024). Developing public health surveillance dashboards: a scoping review on the design principles. BMC. Public Health.

